# Identifying systemic vulnerability phenotypes associated with programmed regimens in frozen embryo transfer: a secondary analysis of a multicentre randomized clinical trial

**DOI:** 10.3389/fmed.2026.1871107

**Published:** 2026-09-03

**Authors:** Junfeng Chen, Hu Li, Mengfei Wang, Bailian Cai, Junjie Qu, Yiran Li

**Affiliations:** 1 Shanghai Key Laboratory of Maternal Fetal Medicine, Shanghai Institute of Maternal-Fetal Medicine and Gynecologic Oncology, Shanghai First Maternity and Infant Hospital, School of Medicine, Tongji University, Shanghai, China; 2 Department of Gynecology, Shanghai First Maternity and Infant Hospital, School of Medicine, Tongji University, Shanghai, China; 3 Center for Reproductive Medicine, Shanghai First Maternity and Infant Hospital, School of Medicine, Tongji University, Shanghai, China

**Keywords:** frozen embryo transfer, machine learning, precision medicine, pre-eclampsia, treatment effects

## Abstract

**Background:**

Programmed endometrial preparation is widely used for frozen embryo transfer (FET) because it allows flexible scheduling. Recent randomized evidence has shown that programmed regimens increase the risk of hypertensive disorders of pregnancy, particularly pre-eclampsia, compared with natural ovulation regimens. However, it remains unclear whether this risk is distributed uniformly across patients or concentrated in women with specific baseline characteristics.

**Objective:**

This study aimed to evaluate the heterogeneous treatment effects of programmed regimens versus natural ovulation regimens on pre-eclampsia after FET and to identify baseline phenotypes associated with increased vulnerability across multiple adverse obstetric outcomes.

**Methods:**

We performed an exploratory secondary analysis of a multicentre, open-label randomized clinical trial comparing programmed and natural ovulation regimens for endometrial preparation before FET. The analysis was restricted to women with regular menstrual cycles who achieved clinical pregnancy after FET. The primary outcome was pre-eclampsia. Secondary outcomes included late-onset pre-eclampsia, placenta accreta spectrum, postpartum haemorrhage and caesarean delivery. Inverse probability of treatment weighting was used to improve covariate balance. Individualized treatment effects were estimated using causal forests. Restricted cubic spline models were used as a sensitivity analysis to examine statistical interactions between treatment regimen and key baseline variables.

**Results:**

Among 2,541 women included in the analytical cohort, 1,261 were assigned to natural ovulation and 1,280 to programmed treatment. Pre-eclampsia occurred in 38 women (3.01%) in the natural ovulation group and 60 women (4.69%) in the programmed group, corresponding to an unadjusted absolute risk difference of 1.67 percentage points. The causal forest estimated positive CATEs for all participants (100.0%), with a median estimated absolute risk increase of 1.51 percentage points (IQR, 1.37–1.83). BMI, AMH, and the retrieval-to-randomization interval were the leading contributors to model-estimated treatment-effect heterogeneity. Conventional regression analysis supported a statistically significant treatment interaction for BMI (*P* for interaction = 0.039), whereas interactions involving AMH and the retrieval-to-randomization interval were not statistically significant.

**Conclusion:**

Programmed FET regimens were associated with a higher absolute risk of pre-eclampsia in the pregnancy-conditioned analytical cohort, although the estimated magnitude of this increase varied across participants. BMI showed the strongest support as a treatment-effect modifier in conventional regression analysis, whereas AMH and the retrieval-to-randomization interval should be interpreted as exploratory candidate modifiers identified by causal forest analysis. These findings require prospective validation before they can be used to guide individualized regimen selection.

## 1 Introduction

The use of frozen embryo transfer (FET) has increased rapidly over the past decade (1, 2). This change has been driven by advances in vitrification, broader adoption of freeze-all strategies and increasing use of preimplantation genetic testing (3). As FET has become a central component of assisted reproductive technology, the choice of endometrial preparation regimen has gained clinical importance (2, 4). The goal is not limited to achieving implantation and pregnancy. Maternal and neonatal safety must also be considered when selecting a regimen (3).

Among women with regular ovulatory cycles, natural ovulation regimens and programmed regimens are the two most frequently used approaches (5). Natural ovulation regimens rely on endogenous follicular development, ovulation and corpus luteum function (6). Programmed regimens use exogenous oestrogen and progesterone to prepare the endometrium and usually suppress spontaneous ovulation (5). Programmed cycles are convenient because they allow flexible scheduling of embryo transfer and simplify clinical workflow. Despite this advantage, accumulating evidence has raised concerns about their obstetric safety.

The PnROVE trial provided important randomized evidence showing that programmed regimens increase the risk of hypertensive disorders of pregnancy, especially pre-eclampsia, compared with natural ovulation regimens (7). A plausible explanation is related to the absence of the corpus luteum in programmed cycles. The corpus luteum produces progesterone and several vasoactive mediators, including relaxin and vascular endothelial growth factor (8). These factors support early maternal vascular adaptation and contribute to trophoblast invasion of maternal spiral arteries (8). When the corpus luteum is absent, early vascular and placental adaptation may be impaired, which may increase the risk of pre-eclampsia and other placental disorders (9).

Although the average treatment effect of programmed regimens has been established, this estimate does not fully address clinical decision-making at the individual level. In practice, complete avoidance of programmed cycles may not be feasible. Some patients have subtle ovulatory dysfunction, irregular scheduling constraints or other clinical reasons that make a natural cycle difficult (10). Therefore, the key question is not only whether programmed regimens increase risk on average, but also which patients are most susceptible to this risk (11).

Traditional regression models are useful for estimating average associations and testing selected interactions (12). However, they are less suited to identifying complex, non-linear and high-dimensional patterns of treatment effect heterogeneity (13). Causal machine learning offers a complementary approach by estimating individualized or conditional treatment effects while allowing flexible interactions among baseline variables (14, 15).

In this exploratory secondary analysis of the publicly available, de-identified PnROVE trial dataset, we evaluated whether the adverse obstetric effect of programmed FET regimens differs according to baseline clinical characteristics. We aimed to estimate the individualized treatment effect of programmed regimens on pre-eclampsia, identify the main clinical modifiers of this effect and determine whether these modifiers also contribute to heterogeneity across other adverse pregnancy outcomes.

## 2 Methods

### 2.1 Study design and participants

This study was an exploratory secondary analysis of the PnROVE trial, a multicentre, open-label randomized controlled trial that compared natural ovulation and programmed regimens for endometrial preparation before FET in ovulatory women (7). Details of the original trial design, eligibility criteria and primary results have been published previously. The original trial was conducted in accordance with the Declaration of Helsinki and was approved by the institutional review boards of participating reproductive centres. All participants provided written informed consent before enrolment. Because the present analysis used a fully de-identified and publicly accessible dataset, additional institutional ethical approval was not required.

For this secondary analysis, we included women who achieved clinical pregnancy after FET and had sufficient follow-up for assessment of mid- and late-pregnancy complications. The derivation of the final analytical cohort is presented in Supplementary Figure S1. Because clinical pregnancy occurs after randomization, this restriction defines a pregnancy-conditioned analytical cohort rather than the original intention-to-treat population. Therefore, the estimated treatment effects should be interpreted as conditional treatment effects among women who achieved clinical pregnancy after FET. Women randomized to a programmed regimen were included in the intervention group, and those randomized to a natural ovulation regimen were included in the control group. To preserve the temporal structure required for causal inference, variables measured after assignment of the endometrial preparation regimen were not included as baseline covariates. Endometrial thickness on the day of transfer and early implantation-related variables were thus excluded from the covariate matrix.

### 2.2 Outcomes

The primary outcome was pre-eclampsia. It was defined according to international obstetric criteria as new-onset hypertension after 20 weeks of gestation accompanied by proteinuria or evidence of maternal end-organ dysfunction (16, 17). Secondary outcomes were selected to assess whether the identified treatment effect heterogeneity was limited to hypertensive disorders or reflected broader disruption of maternal-fetal adaptation. These outcomes included late-onset pre-eclampsia, placenta accreta spectrum, postpartum haemorrhage and caesarean delivery (18).

### 2.3 Covariates

Baseline covariates were selected before outcome modelling and were limited to variables measured before assignment of the FET regimen. These included maternal age at FET, body mass index (BMI), anti-Müllerian hormone (AMH), duration of infertility, interval from oocyte retrieval to randomization, gravidity, parity, history of caesarean delivery, previous transfer failure and embryonic developmental stage. These variables were chosen because of their potential relevance to reproductive prognosis, placental development or treatment selection.

### 2.4 Statistical analysis

Participants with missing values in key baseline covariates or the primary outcome were excluded from the main analysis. Because clinical pregnancy occurred after treatment assignment, restricting the analysis to women who achieved clinical pregnancy may induce post-randomization selection and collider bias and may have altered the baseline balance established by randomization. Inverse probability of treatment weighting (IPTW) was therefore used only as a supplementary covariate-balancing procedure within the selected pregnancy cohort to reduce measured baseline imbalance between the programmed and natural ovulation groups (19, 20). Although weighting can improve balance in measured baseline covariates, it cannot recover the original intention-to-treat estimand or fully eliminate selection bias arising from unmeasured factors related to pregnancy achievement or outcome risk. The propensity score for receiving a programmed regimen was estimated using multivariable logistic regression with the prespecified baseline covariates. Covariate balance before and after weighting was assessed using standardized mean differences. A standardized mean difference below 0.1 was considered to indicate adequate balance. The IPTW weights were used to assess and improve measured covariate balance and were not supplied as external sample weights to the causal forest models.

As a sensitivity analysis, we used multivariable logistic regression with restricted cubic splines to evaluate whether the relationship between treatment regimen and pre-eclampsia varied across key continuous baseline variables. Interaction terms between treatment regimen and the main variables identified by the causal forest model were examined. This approach was used to provide a conventional statistical comparison with the machine learning findings.

### 2.5 Causal machine learning analysis

Heterogeneous treatment effects were estimated using causal forests. This non-parametric method estimates conditional average treatment effects while allowing flexible and non-linear interactions between treatment assignment and baseline characteristics. The method also uses orthogonalization to reduce the influence of confounding from observed covariates. In this study, the conditional average treatment effect was interpreted as the individualized absolute risk difference associated with programmed regimens compared with natural ovulation regimens.

For the primary outcome, a causal forest model with 2,000 trees and tuned hyperparameters was fitted. The distribution of predicted individualized treatment effects was examined to evaluate whether programmed regimens increased or decreased absolute risk across participants. Variable importance scores were extracted to identify the baseline characteristics that contributed most to treatment effect heterogeneity. Partial dependence plots with locally estimated smoothing were used to describe the direction and pattern of effect modification.

To evaluate whether the same modifiers were relevant to other obstetric outcomes, the causal forest pipeline was repeated for each secondary outcome. Variable importance scores from these models were summarized across outcomes to identify baseline characteristics that consistently modified the effect of programmed regimens.

All analyses were performed using R version 4.6.1. Causal forest analyses were implemented using the grf package (version 2.6.1). Inverse probability of treatment weighting and covariate-balance assessment were conducted using WeightIt (version 1.7.0) and cobalt (version 4.6.3), respectively. Restricted cubic spline analyses were performed using rms (version 8.1.1). Data processing and visualization were conducted using tidyverse (version 2.0.0), ggplot2 (version 4.0.3), patchwork (version 1.3.2), and reshape2 (version 1.4.5). No additional cross-validation or repeated-resampling stability analysis was performed.

## 3 Results

### 3.1 Analytical cohort, outcome events and covariate balance

**Figure 1:**
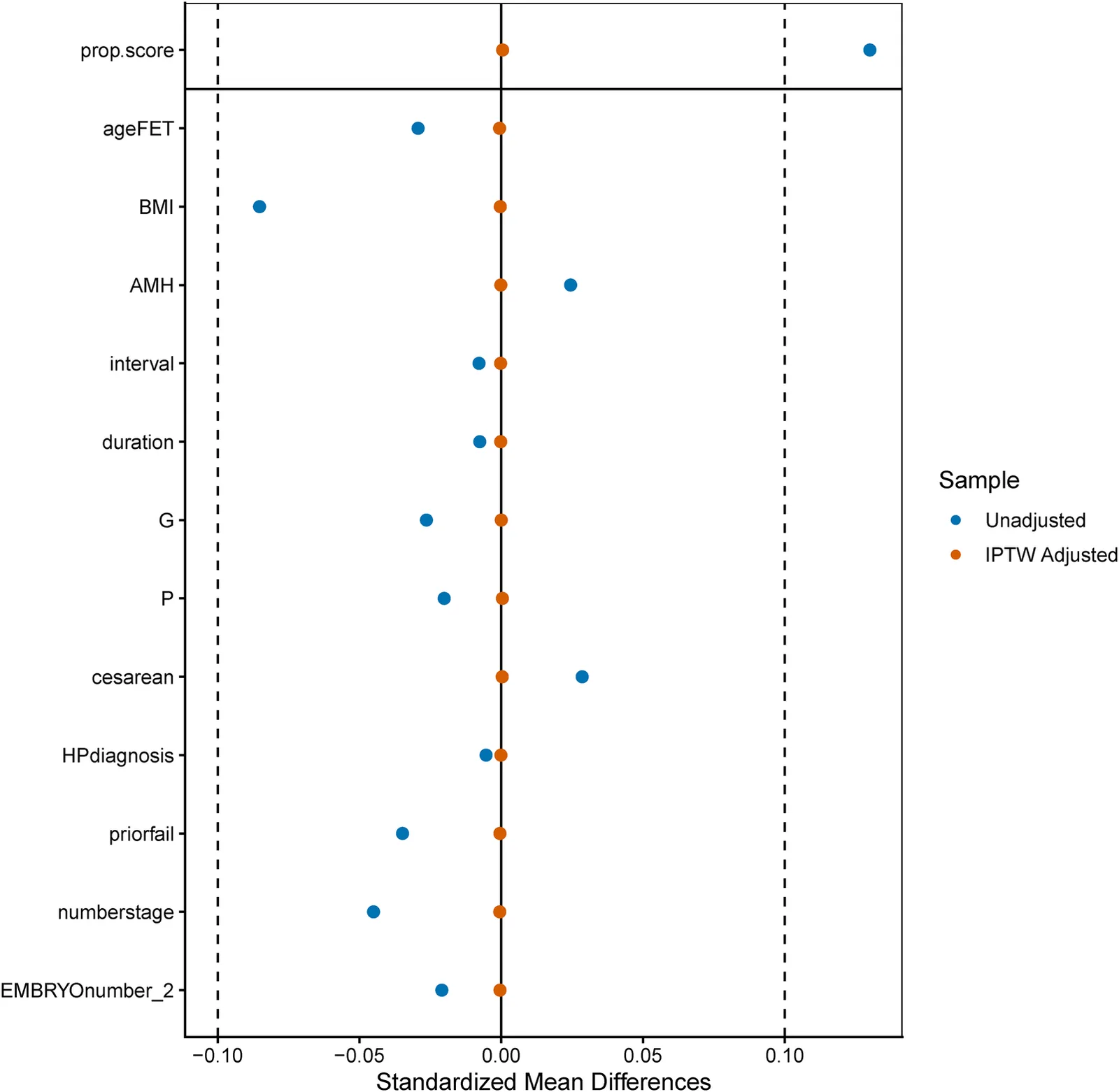
Covariate balance before and after inverse probability of treatment weighting (IPTW). Standardised mean differences (SMD) for baseline characteristics between the programmed and natural cycle groups. All covariates achieved optimal balance post-weighting (SMD < 0.1).

Of the 4,376 participants randomized in the PnROVE trial, 2,628 achieved clinical pregnancy. After excluding 87 participants with missing data for at least one prespecified baseline covariate, 2,541 participants were included in the primary analytical cohort, comprising 1,261 women assigned to the natural ovulation regimen and 1,280 assigned to the programmed regimen (Supplementary Figure S1). Several baseline variables showed minor imbalance before weighting. After IPTW, all measured covariates were balanced between the programmed and natural ovulation groups, with standardized mean differences below 0.1 (Figure 1). This balance provided a suitable basis for estimating treatment effects within the pregnancy-only cohort. Among the 2,541 women included in the primary analytical cohort, pre-eclampsia occurred in 98 women overall (3.86%). The observed event rate was 38 of 1,261 women (3.01%) in the natural ovulation group and 60 of 1,280 women (4.69%) in the programmed group, corresponding to an unadjusted absolute risk difference of 1.67 percentage points.

### 3.2 Individualized treatment effects for pre-eclampsia

**Figure 2:**
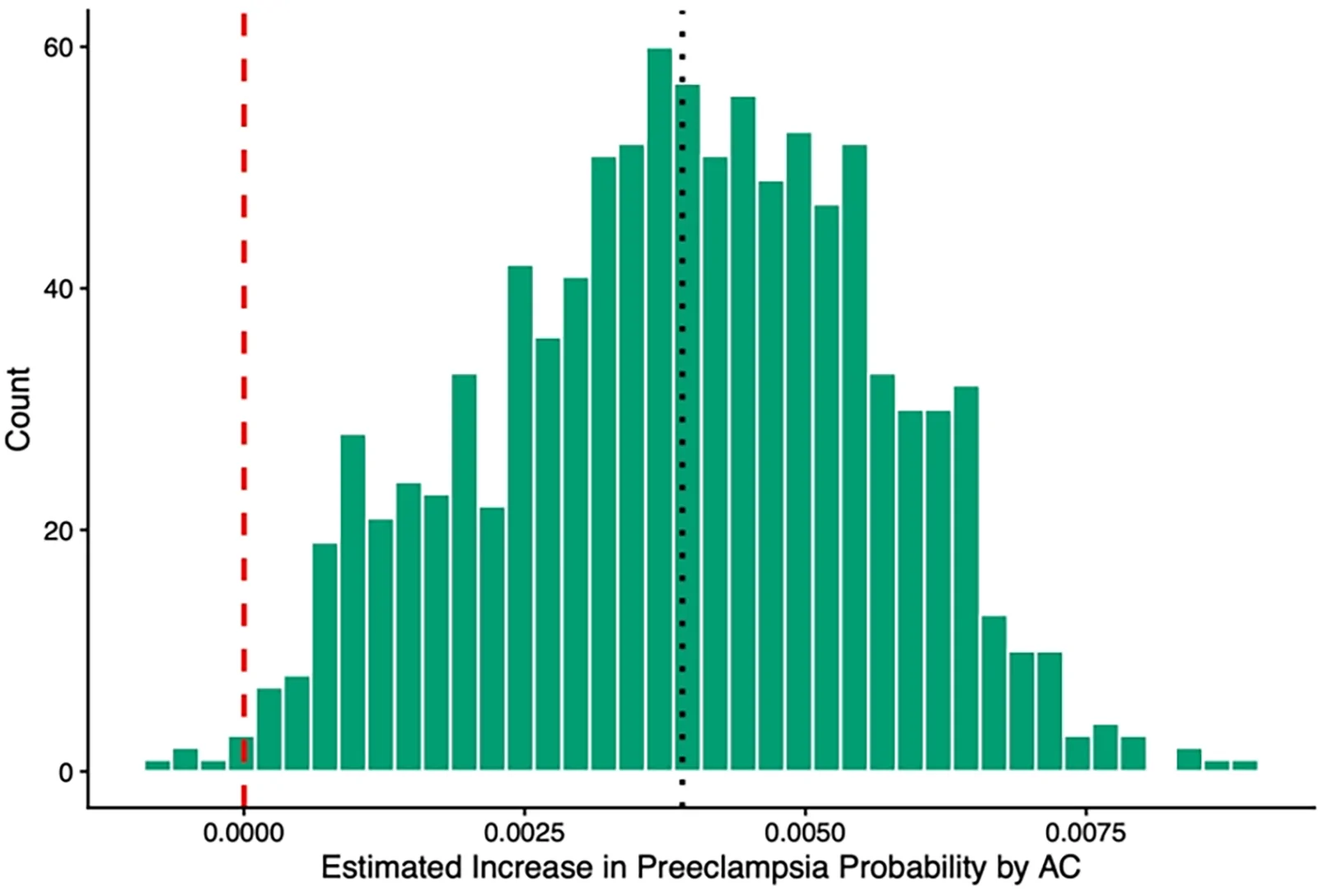
Distribution of individualized treatment effects on pre-eclampsia. Histogram displaying the conditional average treatment effects (CATE) estimated by causal forests. The distribution consistently falls to the right of the zero-harm threshold (red dashed line), indicating a universal but highly heterogeneous absolute risk increase associated with programmed cycles.

The causal forest estimated positive CATEs for all 2,541 participants (100.0%), indicating that the model-predicted direction of the treatment effect was consistently toward higher pre-eclampsia risk with programmed regimens. The mean estimated absolute risk increase was 1.59 percentage points, the median was 1.51 percentage points, and the interquartile range was 1.37–1.83 percentage points. The estimated CATEs ranged from 1.05 to 2.18 percentage points, showing that the direction of the model-estimated risk increase was consistent, whereas its magnitude varied across participants (Figure 2).

### 3.3 Baseline modifiers of treatment effect

**Figure 3:**
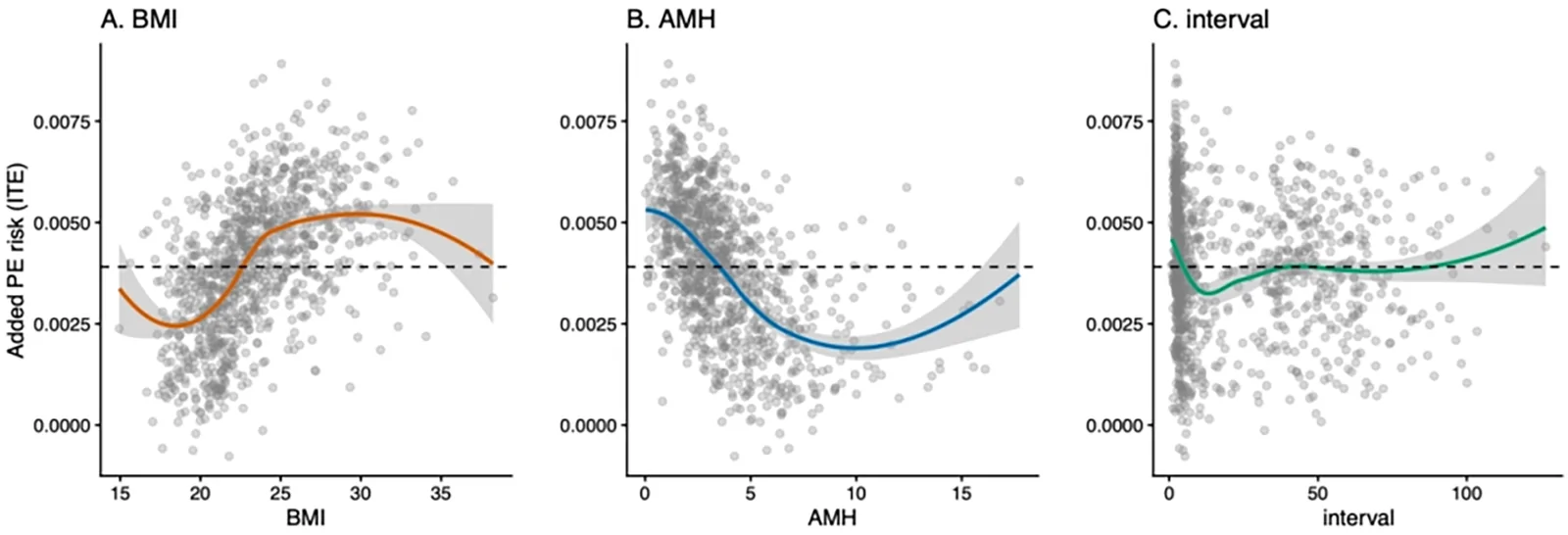
Non-linear dependence of programmed cycle risk on top baseline features. Partial dependence plots (with LOESS smoothing and 95% CIs) illustrating the modifying effects of **(A)** BMI, **(B)** AMH, and **(C)** retrieval-to-randomisation interval on the absolute risk increase of pre-eclampsia.

Variable importance analysis identified BMI, AMH and the interval from oocyte retrieval to randomization as the main contributors to treatment effect heterogeneity (Table 1). These variables had greater influence than chronological maternal age in the causal forest model. Partial dependence analysis suggested variation in the model-estimated treatment effect across BMI, AMH, and retrieval-to-randomization interval values (Figure 3). Because no clinical BMI threshold for regimen selection was prespecified or validated in the present analysis, BMI was evaluated across its observed distribution rather than categorized as “optimal” or “non-optimal.” Across BMI quartiles of 14.87–20.53, >20.53–22.41, >22.41–24.78, and >24.78–38.57 kg/m^2^, the mean estimated CATEs were 1.60, 1.60, 1.59, and 1.56 percentage points, respectively. These model-derived patterns suggest modest BMI-related treatment-effect heterogeneity but do not establish a clinically actionable BMI cutoff. Lower AMH levels and shorter retrieval-to-randomization intervals were associated with larger model-estimated absolute risk increases. Across AMH quartiles, the mean estimated CATE decreased from 1.65 percentage points in the lowest quartile to 1.52 percentage points in the highest quartile. Similarly, across retrieval-to-randomization interval quartiles, the mean estimated CATE decreased from 1.89 percentage points in the shortest-interval quartile to 1.36 percentage points in the longest-interval quartile. These patterns represent exploratory causal-forest signals rather than formally confirmed effect modification. The sensitivity analysis using restricted cubic splines provided conventional statistical support only for the modifying role of BMI (Supplementary Figure S2). The interaction between treatment regimen and BMI was statistically significant (*P* for interaction = 0.039). In contrast, interactions involving AMH, the retrieval-to-randomization interval, and maternal age did not reach conventional statistical significance. Therefore, although AMH and the retrieval-to-randomization interval contributed to heterogeneity in the causal forest model, they should not be interpreted as statistically confirmed effect modifiers in the conventional regression framework.

**Table 1:** Variable importance for heterogeneity analysis.

Variable	Importance
BMI	0.224
AMH	0.206
Interval	0.167
AgeFET	0.135
Priorfail	0.082
Duration	0.064
P	0.031
G	0.026
Numberstage	0.012
Cesarean	0.008
HPdiagnosis	0.000
EMBRYOnumber	0.000

Variable importance represents the relative contribution of each baseline characteristic to explaining heterogeneity in estimated treatment effects within the causal forest model. It does not represent an independent causal effect of the variable itself, nor does it provide a formal statistical significance test.

### 3.4 Clinical risk profiles

To translate individualized predictions into clinically interpretable profiles, participants were stratified into quartiles according to their predicted treatment-related increase in pre-eclampsia risk. Women in the highest-risk quartile showed a distinct clinical profile compared with those in the lowest-risk quartile (Table 2). The high-risk group was characterized by higher BMI, lower AMH and a shorter interval from oocyte retrieval to transfer-related randomization.

**Table 2:** Characteristics and outcome rates in high- and low-risk subgroups.

Risk_Label	Mean_ageFET	Mean_BMI	Mean_AMH	Mean_interval	Mean_duration	PE_Rate_AC	PE_Rate_NC
High AC-Harm (Top 25%)	34.265	25.577	2.325	19.455	3.298	2.326	8.943
Low AC-Harm (Bottom 25%)	32.307	21.034	5.664	24.743	3.665	8.571	0.885

Unadjusted event rates in these subgroups suggested potential confounding by indication. In the algorithmically defined high-risk subgroup, crude pre-eclampsia rates were higher in the natural ovulation group than in the programmed group. This pattern likely reflects clinical selection of patients with poorer baseline prognosis into different regimens. After causal adjustment, the model indicated that replacing a natural ovulation regimen with a programmed regimen would further increase absolute risk in this vulnerable subgroup.

### 3.5 Multi-outcome analysis

**Figure 4:**
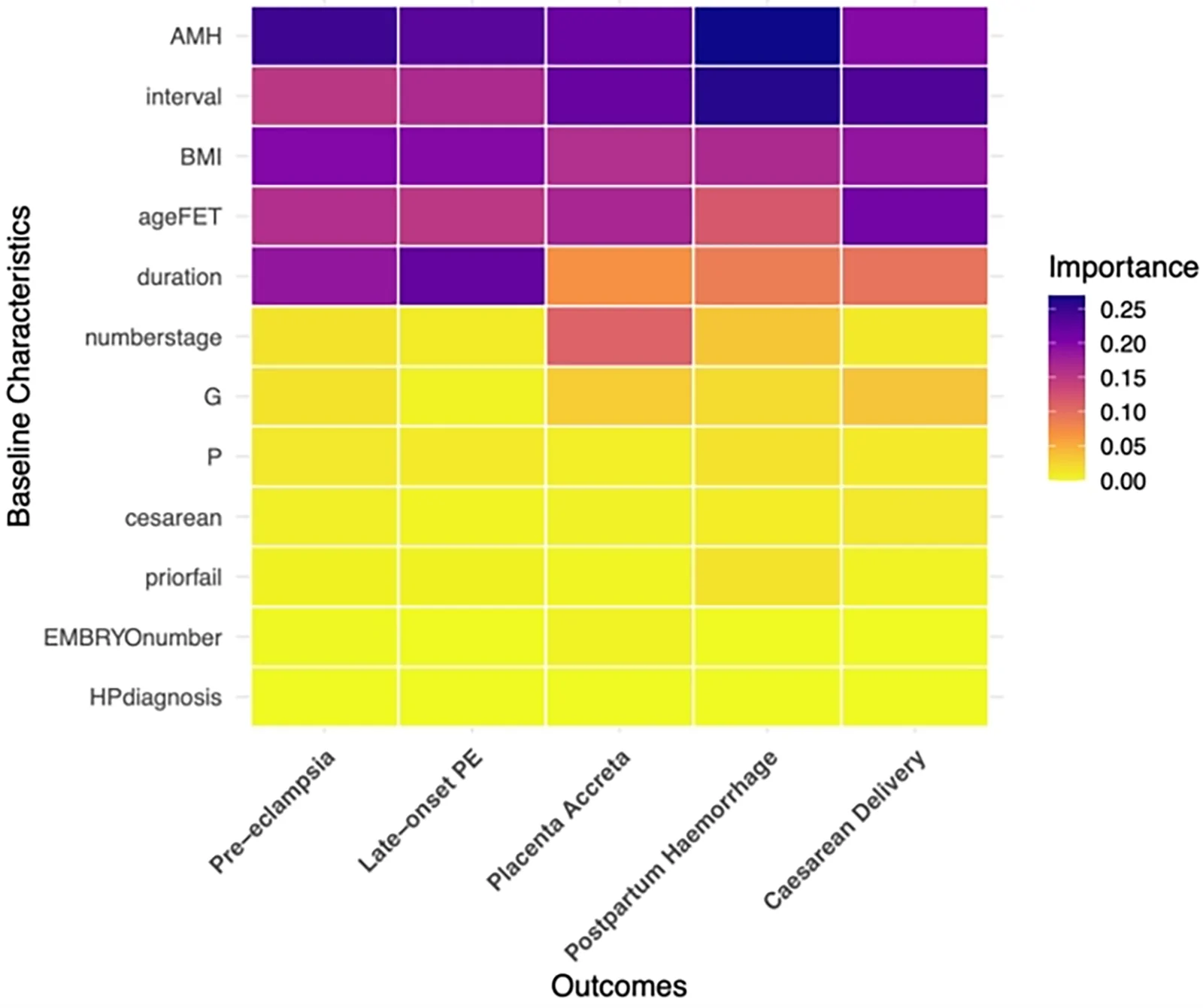
Pan-complication heatmap of common vulnerability phenotypes. Variable importance scores extracted from multi-outcome causal forests.

The multi-outcome causal analysis showed consistent patterns across adverse obstetric outcomes (Figure 4 and Table 3). BMI, AMH and retrieval-to-randomization interval remained important contributors to model-estimated treatment-effect heterogeneity not only for pre-eclampsia but also for late-onset pre-eclampsia, placenta accreta spectrum, postpartum haemorrhage and caesarean delivery. This consistency suggests that these baseline characteristics may define a broader vulnerability phenotype rather than a risk profile limited to hypertensive disorders.

**Table 3:** Variable importance across pregnancy-related complications.

Variable	Pre-eclampsia	Late-onset PE	Placenta accreta	Postpartum haemorrhage	Caesarean delivery	Average_Importance
AMH	0.247	0.230	0.218	0.269	0.198	0.232
Interval	0.153	0.167	0.219	0.261	0.236	0.207
BMI	0.199	0.198	0.161	0.168	0.188	0.183
AgeFET	0.160	0.150	0.170	0.120	0.210	0.162
Duration	0.187	0.223	0.070	0.088	0.096	0.133
Numberstage	0.015	0.009	0.111	0.035	0.010	0.036
G	0.015	0.004	0.030	0.020	0.036	0.021
P	0.012	0.010	0.008	0.016	0.010	0.011
Cesarean	0.006	0.004	0.005	0.008	0.012	0.007
Priorfail	0.005	0.005	0.003	0.015	0.004	0.006
EMBRYOnumber	0.001	0.000	0.004	0.000	0.000	0.001
HPdiagnosis	0.000	0.000	0.000	0.000	0.000	0.000

## 4 Discussion

In this exploratory secondary analysis of a multicentre randomized clinical trial, programmed FET regimens were associated with a higher observed absolute risk of pre-eclampsia than natural ovulation regimens. Importantly, the estimated magnitude of the treatment-associated increase in absolute risk varied across participants. BMI, AMH and the interval from oocyte retrieval to randomization were the leading candidate contributors to model-estimated treatment-effect heterogeneity and were also relevant to several other adverse obstetric outcomes.

These findings extend previous trial-level evidence by moving from average treatment effects to individualized risk estimation. The original randomized evidence showed that programmed regimens increase the overall risk of hypertensive disorders of pregnancy. Our analysis suggests that this risk may be particularly important in women with specific metabolic and ovarian reserve profiles. This distinction is clinically relevant because treatment decisions in FET are made for individual patients rather than for an average population.

The observed vulnerability pattern is biologically plausible. In natural ovulation cycles, the corpus luteum produces vasoactive and angiogenic mediators that support early maternal cardiovascular adaptation and placental development (21–23). In programmed cycles, the absence of a corpus luteum may impair these processes (24, 25). Women with higher metabolic burden or altered ovarian reserve markers may have reduced capacity to compensate for this endocrine and vascular deficit (26, 27). Under these conditions, programmed regimens may contribute to impaired decidualization, abnormal trophoblast invasion and subsequent obstetric complications.

BMI was the most consistently validated modifier. The restricted cubic spline analysis confirmed a significant interaction between BMI and treatment regimen. This result supports the clinical relevance of metabolic status when choosing an endometrial preparation protocol. AMH and retrieval-to-randomization interval did not show significant two-way interactions in conventional regression models, but they were important in causal forest analysis and showed consistent signals across outcomes. The difference between causal forest and regression-based interaction analyses is expected because the two approaches address different analytical questions. Conventional interaction models usually evaluate prespecified marginal interactions, whereas causal forests identify treatment-effect heterogeneity through flexible combinations of multiple covariates. Therefore, variables with high causal-forest importance but non-significant marginal interactions may still contribute to multivariable heterogeneity patterns, although they should not be interpreted as formally confirmed effect modifiers.

The multi-outcome analysis is another important aspect of this study. The same three baseline variables were repeatedly identified across hypertensive, placental and delivery-related outcomes. This pattern suggests that programmed regimens may not simply increase one isolated complication. Instead, in susceptible patients, the absence of corpus luteum-derived factors may contribute to a broader disturbance in maternal-fetal adaptation. The term systemic vulnerability phenotype is therefore used here as a descriptive framework to summarize a reproducible pattern of baseline characteristics associated with model-estimated treatment-effect heterogeneity. Although this phenotype may help clinicians recognize patients who appear more susceptible to programmed-cycle-associated obstetric risk, it should be regarded as a hypothesis-generating construct rather than a ready-to-use decision rule. The BMI, AMH, and retrieval-to-randomization interval patterns observed in this study do not define validated clinical thresholds for selecting endometrial preparation regimens. Before this approach can be incorporated into routine practice, the proposed vulnerability phenotype requires external validation in independent FET cohorts, assessment of model calibration and clinical utility, and evaluation alongside the probability of achieving pregnancy with each regimen.

The findings may have practical implications. For women with a high predicted vulnerability profile, natural ovulation or modified natural regimens may be preferable when clinically feasible. When a programmed regimen is necessary, these patients may benefit from closer obstetric surveillance and careful counselling regarding potential risks. The results also provide a framework for developing decision-support tools that incorporate baseline metabolic, ovarian reserve and timing-related variables into FET planning.

This study has several strengths. It used data from a multicentre randomized trial, which reduced many sources of bias commonly seen in observational studies. IPTW improved measured covariate balance in the pregnancy-only analytical cohort. The combination of causal forests and conventional interaction modelling allowed flexible exploration of treatment effect heterogeneity while retaining statistical interpretability (28, 29). The extension to multiple adverse outcomes also provided a broader view of maternal safety.

Several limitations should be considered. First, this was an exploratory secondary analysis, and the findings should be regarded as hypothesis-generating. In addition, because the analysis was restricted to women who achieved clinical pregnancy, conditioning on this post-randomization event may have introduced selection or collider bias. Although IPTW improved balance of measured baseline characteristics within the selected cohort, it cannot completely eliminate bias related to unmeasured factors affecting pregnancy achievement. Prospective validation in independent cohorts is needed before the phenotype can be used for routine clinical decision-making. Second, causal machine learning depends on the assumption that all relevant confounders were measured and appropriately included. Unmeasured genetic, immunological, paternal or treatment-related factors may still influence the observed heterogeneity. Third, intermediate variables such as endometrial thickness were excluded to avoid overadjustment. This approach was appropriate for estimating treatment effects, but it limited assessment of downstream biological mediators. Finally, some secondary outcomes may have limited event numbers, which can affect the stability of variable importance estimates.

## 5 Conclusion

Programmed regimens for frozen embryo transfer were associated with a higher absolute risk of pre-eclampsia in the pregnancy-conditioned analytical cohort, although the model-estimated magnitude of this increase varied across participants. BMI showed a statistically significant interaction with treatment regimen in conventional regression analysis, whereas AMH and the retrieval-to-randomization interval emerged as candidate contributors to treatment-effect heterogeneity in the causal forest analysis but were not statistically confirmed as effect modifiers. These exploratory findings require prospective and external validation before they can inform individualized selection of endometrial preparation regimens.

## Data Availability

The original contributions presented in the study are included in the article/Supplementary Material, further inquiries can be directed to the corresponding authors.
