# Supplementary material for: Identifying systemic vulnerability phenotypes associated with programmed regimens in frozen embryo transfer: a secondary analysis of a multicentre randomized clinical trial
Source: Front Med (Lausanne). 2026 Sep 3;13:1871107. doi: 10.3389/fmed.2026.1871107 (PMC13581899; doi:10.3389/fmed.2026.1871107)
Supplement: Supplementary file 1 [file Supplementaryfile1.docx]

**Figure S1.**

Figure S1. Flow diagram of participant selection and analytical cohort derivation.

**Figure S2.**

Figure S2. Model-predicted probabilities of pre-eclampsia are shown according to (A) maternal body mass index (BMI), (B) anti-Müllerian hormone (AMH) level, (C) the interval from oocyte retrieval to randomization, and (D) maternal age at frozen embryo transfer (FET), stratified by endometrial preparation regimen.
